# Ectopic Catalase Expression in Mitochondria by Adeno-Associated Virus Enhances Exercise Performance in Mice

**DOI:** 10.1371/journal.pone.0006673

**Published:** 2009-08-19

**Authors:** Dejia Li, Yi Lai, Yongping Yue, Peter S. Rabinovitch, Chady Hakim, Dongsheng Duan

**Affiliations:** 1 Department of Molecular Microbiology and Immunology, School of Medicine, University of Missouri, Columbia, Missouri, United States of America; 2 Department of Pathology, University of Washington, Seattle, Washington, United States of America; Universidad Europea de Madrid, Spain

## Abstract

Oxidative stress is thought to compromise muscle contractility. However, administration of generic antioxidants has failed to convincingly improve performance during exhaustive exercise. One possible explanation may relate to the inability of the supplemented antioxidants to effectively eliminate excessive free radicals at the site of generation. Here, we tested whether delivering catalase to the mitochondria, a site of free radical production in contracting muscle, could improve treadmill performance in C57Bl/6 mice. Recombinant adeno-associated virus serotype-9 (AV.RSV.MCAT) was generated to express a mitochondria-targeted catalase gene. AV.RSV.MCAT was delivered to newborn C57Bl/6 mouse circulation at the dose of 10^12^ vector genome particles per mouse. Three months later, we observed a ∼2 to 10-fold increase of catalase protein and activity in skeletal muscle and the heart. Subcellular fractionation western blot and double immunofluorescence staining confirmed ectopic catalase expression in the mitochondria. Compared with untreated control mice, absolute running distance and body weight normalized running distance were significantly improved in AV.RSV.MCAT infected mice during exhaustive treadmill running. Interestingly, ex vivo contractility of the extensor digitorum longus muscle was not altered. Taken together, we have demonstrated that forced catalase expression in the mitochondria enhances exercise performance. Our result provides a framework for further elucidating the underlying mechanism. It also raises the hope of applying similar strategies to remove excessive, pathogenic free radicals in certain muscle diseases (such as Duchenne muscular dystrophy) and ameliorate muscle disease.

## Introduction

It has long been recognized that muscle activity is tightly regulated by free radicals (reviewed in [1]–[3]). Free radicals are short-lived, highly reactive molecules. Low levels of free radicals are required for normal muscle contraction and metabolism (reviewed in [1], [4]). However, untempered free radical production during strenuous exercise results in muscle fatigue and reduces performance [5], [6]. It has been hypothesized that exogenous antioxidant supplementation may help scavenge excessive free radicals and improve muscle performance during exercise. Yet, this claim has not been substantiated by clinical studies (reviewed in [3], [7]–[11]).

The lack of performance enhancement by generic antioxidants is reminiscent of a similar observation in aging studies. Oxidative stress has been considered as a key determinant of the lifespan in drosophila and C. elegans [12], [13]. However, mouse studies have yielded conflicting results (reviewed in [14]–[17]). Of particular interests are these performed in catalase transgenic mice. Catalase is a major cellular antioxidant enzyme normally expressed in the peroxisomes (abbreviated as PCAT in this manuscript). Transgenic over-expression of catalase in the peroxisome or nucleus did not extend mouse lifespan [18]–[20]. However, targeting catalase to the mitochondria resulted in a 20% lifespan increase in transgenic mice [20]. Furthermore, these mice have enhanced retention of cardiac performance with age [21]. These findings reveal the importance of subcellular antioxidant expression on the functional outcome. Here, we hypothesize that targeted catalase expression in the mitochondria can enhance exercise performance in mice. To test this hypothesis, we engineered the mitochondrial-targeted catalase gene (MCAT) in serotype-9 recombinant adeno-associated viral vector (AAV-9). After systemic delivery in newborn C57Bl/6 (BL6) mice, we confirmed ectopic mitochondrial catalase expression. At the three months of the age, we examined exercise performance. In support of our hypothesis, running distance was significantly increased in AAV infected mice. Interestingly, mitochondrial targeted catalase expression did not alter the contractile profile in the isolated extensor digitorum longus (EDL) muscle.

## Results

### Characterization of ectopic catalase expression in the mitochondria after systemic AAV-9 delivery in neonatal mice

**Figure 1:**
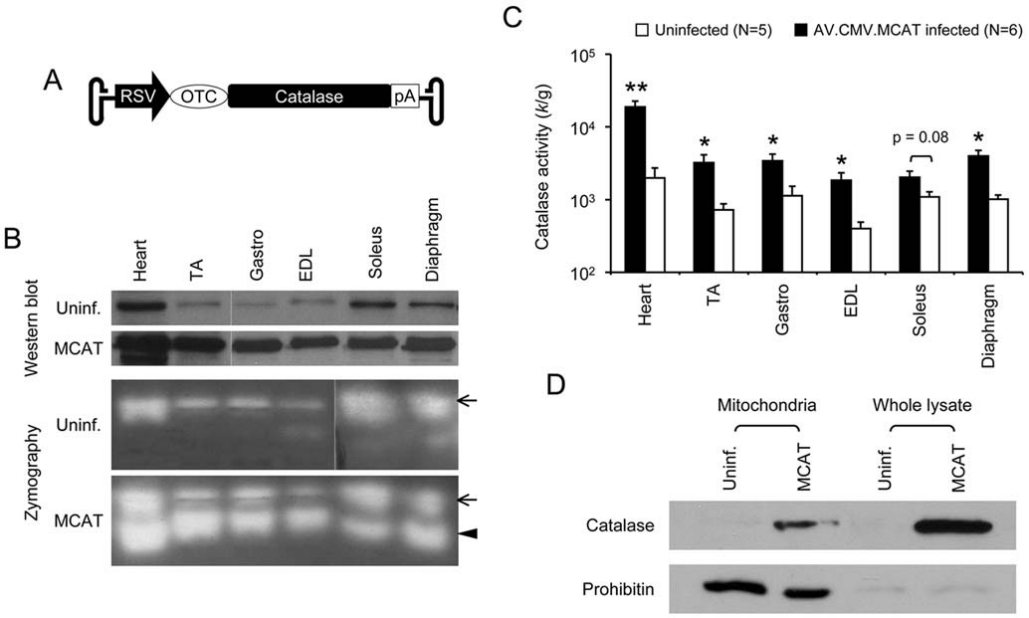
Characterization of AV.RSV.MCAT vector. A, Schematic outline of the AAV vector. The flanking hairpin structures denote AAV inverted terminal repeats. RSV, Rose sarcoma virus promoter; OTC, mitochondrial targeting sequence from the ornithine transcarbamylase gene; pA, polyadenylation signal from SV40 virus. Not drawn to scale. B, Determination of catalase expression from AAV infected mice by whole muscle lysate western blot and the in-gel zymography assay. Photomicrographs are the representative results from three independent experiments. Uninf., mice not infected by AV.RSV.MCAT. MCAT, mice received systemic AV. RSV.MCAT infection. Dotted lines, images were spliced together from the same gel but were run on noncontiguous lanes. Arrow, endogenous murine catalase; Arrowhead, mitochondrial expressed human catalase from AAV vector. TA, tibialis anterior muscle; Gastro, gastrocnemius muscle, EDL, extensor digitorium longus muscle. C, Catalase activity in whole muscle lysate. Asterisk, significantly higher than that of the uninfected group (p<0.05); Double asterisks, significantly higher than that of the uninfected group (p<0.005). D, Western blot analysis of mitochondrial and whole muscle lysate preparations from the gastrocnemius muscle. Prohibitin is a mitochondria marker. Uninf., mice not infected by AV.RSV.MCAT. MCAT, mice received systemic AV. RSV.MCAT infection.

Recombinant AAV-9 AV.RSV.MCAT vector was generated to express the MCAT gene (Figure 1A). 1×10^12^ vector genome particles of AV.RSV.MCAT were delivered to 2-day-old C57Bl/6 mice through the vasculature as we described before [22], [23]. Consistent with transgenic study [20], AV.RSV.MCAT infected mice showed similar growth rate as uninfected control littermates (data not shown).

**Figure 2:**
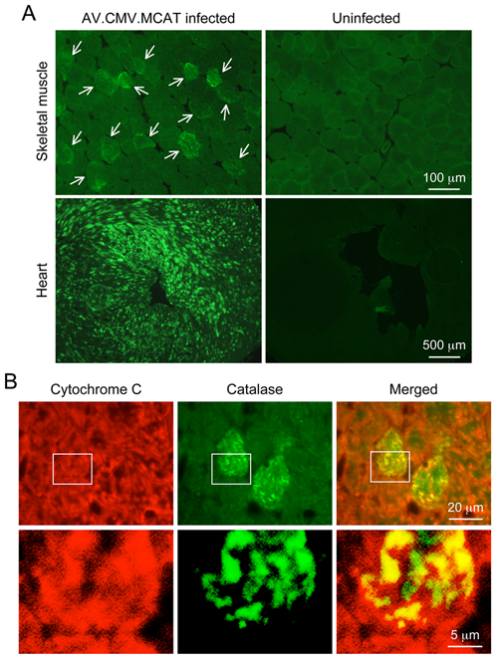
Systemic AV.RSV.MCAT infection leads to mosaic catalase expression in the mitochondria in striated muscles. A, Representative catalase immunofluorescence staining photomicrographs of AV.RSV.MCAT infected and uninfected skeletal muscle (top panels) and heart (bottom panels). Arrow, AAV transduced skeletal muscle myofiber. B, Representative double immunofluorescence staining photomicrographs of AV.RSV.MCAT infected heart. Bottom panels are high magnification images of the boxed region in respective top panels. Cytochrome C marks mitochondria (red color). Catalase is in green color. Yellow color in merged images reveals mitochondrial catalase expression.

Three months after AV.RSV.MCAT infection, we examined catalase expression in skeletal muscle and the heart (Figures 1 and 2). On whole muscle lysate western blot, the intensity of the catalase band was substantially stronger in AV.RSV.MCAT infected mice (Figure 1B). Catalase overexpression was confirmed by the zymograph assay (Figure 1B). Interestingly, for reason(s) yet unknown, MCAT (Figure 1B arrowhead) appeared to migrate faster than endogenous PCAT in the zymography gel (Figure 1B arrows). Next, we quantified catalase activity in whole muscle lysate. Compared with uninfected mice, we observed an approximately 10-fold catalase activity increase in the heart of AV.RSV.MCAT infected mice. In skeletal muscle, the catalase activity was increased by 3 to 5-fold except for the soleus muscle which only showed a less than 2-fold increase (Figure 1C). To confirm mitochondrial catalase expression, we performed western blot using isolated skeletal muscle mitochondria preparation (Figure 1D) [24]. Prohibitin was used as the mitochondria marker. As expected, we observed the catalase band in the mitochondria isolated from AV.RSV.MCAT infected muscle but not from uninfected muscle (Figure 1D).

To further evaluate MCAT expression, we performed immunofluorescence staining on tissue cryosections (Figure 2). In both skeletal muscle and the heart, we observed mosaic MCAT expression (Figure 2A). The mitochondrial localization of MCAT was confirmed by double immunofluorescence staining (Figure 2B). The mitochondria were labeled with the cytochrome C antibody (Figure 2B, red color). Catalase was revealed with a polyclonal antibody (Figure 2B, green color). Merged images clearly demonstrated co-localization of catalase and cytochrome C, suggesting that MCAT was indeed localized in the mitochondria (Figure 2B, yellow color). Interestingly, we also detected some catalase expression outside the mitochondria (Figure 2B, green color in the merged images). This could represent either endogenous PCAT or newly synthesized MCAT yet to be imported into the mitochondria.

### Systemic AAV-9 AV.RSV.MCAT infection enhances treadmill performance

**Figure 3:**
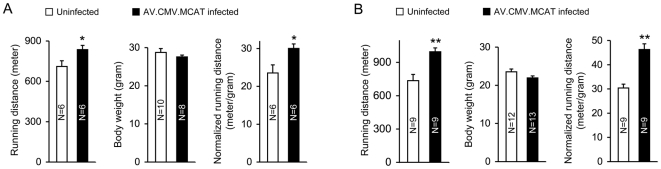
Systemic AV.RSV.MCAT delivery significantly enhances treadmill performance in both male and female mice. A, results from male mice; B, results from female mice. Right panel, absolute running distance; Middle panel, body weight; Left panel, body weight-normalized running distance. Asterisk, significantly different from that of the uninfected group (p<0.04); Double asterisks, significantly higher than that of the uninfected group (p<0.001).

To evaluate the impact on exercise performance, mice were subjected to a single bout of exhaustive treadmill running challenge. In both male and female, AV.RSV.MCAT infection significantly enhanced running performance (Figure 3). Interestingly, the level of improvement was more substantial in female mice. The absolute running distance increased by 18% and 35% in AV.RSV.MCAT infected male and female mice, respectively.

Male body weight was significantly higher than that of female mice (p = 0.013). Next, we compared the body weight-normalized running distance (m/g). In male mice, running distance increased from 23.55±2.14 m/g to 30.04±1.18 m/g (p = 0.0242) (Figure 3A). In female mice, running distance increased from 30.38±1.59 m/g to 46.28±2.37 m/g (p = 0.0004) (Figure 3B).

### Ectopic catalase expression in the mitochondria does not alter contractile property of the isolated EDL muscle

**Figure 4:**
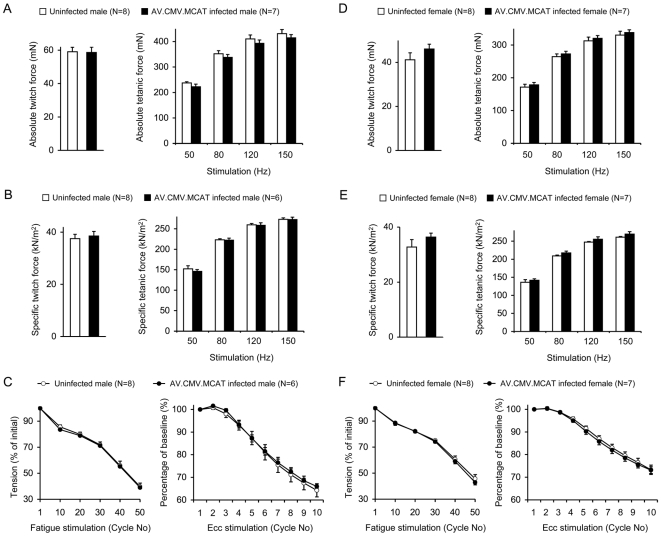
The contractility of the isolated EDL muscle is not altered following systemic AV.RSV.MCAT infection. A to C, results from male mice; D to F, results from female mice. A and D, absolute twitch (left panel) and tetanic forces (right panels). B and E, cross-sectional area normalized specific twitch (left panel) and tetanic forces (right panels). C and F, fatigue response (left panel) and eccentric contraction profile (right panel). Filled bar/circle, AV.RSV.MCAT infected mice; Open bar/circle, uninfected mice.

We compared the anatomic and contractile properties of the freshly isolated EDL muscle. We did not see a significant difference in muscle weight, length and cross-sectional area between AV.RSV.MCAT infected and uninfected mice (Table 1). Specific and absolute muscle forces were not altered either (Figure 4).

**Table 1:** Characterization of the EDL muscle.

Mice	N	Weight (mg)	Lo (mm)	CSA (mm^2^)
Male, no AAV	8	9.53±0.49	12.93±0.23	1.58±0.06
Male, AAV inf	6	8.83±0.18	12.47±0.04	1.52±0.03
Female, no AAV	8	7.19±0.34	12.15±0.26	1.26±0.04
Female, AAV inf	7	7.20±0.14	12.29±0.06	1.26±0.02

We also examined the fatigue and the eccentric contraction responses. 50 cycles of fatigue stimulation were applied to the EDL muscle. Each cycle included 300 ms of 70 Hz stimulation followed by 3 sec rest [25]. In both male and female mice, AV.RSV.MCAT infection did not change the fatigue profile (Figure 4). After the fatigue protocol, muscle was allowed a 15 min rest for recovery. We then applied an eccentric contraction protocol to determine the response to contraction-induced injury [26], [27]. The overall trend of force decline was similar between AV.RSV.MCAT infected and uninfected mice (Figure 4).

Taken together, systemic AV.RSV.MCAT infection induced negligible changes in ex vivo contractility in the isolated EDL muscle.

## Discussion

In this study, we presented evidence that neonatal systemic delivery of an AAV-9 MCAT vector significantly enhances running performance in young adult BL6 mice. Furthermore, ectopic catalase expression in the mitochondria appears to have nominal effect on the contractility of the isolated EDL muscle.

Oxidative stress generated during intensive exercise is thought to compromise physical performance. Surprisingly, administration of generic antioxidants such as vitamin C, vitamine E or β-carotene has failed to convincingly alleviate fatigue and enhance performance in exhaustive exercise (reviewed in [3], [7]–[11]). In contrast, combined administration of vitamin C and vitamin E was recently shown to abolish health-promoting effect of supervised physical training in humans [28]. The disappointing result of antioxidant trials challenges the dogma of the free radical-mediated muscle injury.

It has been well documented in the literature that exercise increases muscle free radical production (reviewed in [5], [6], [29]). More recently, Jackson and colleagues applied in vivo microdialysis technique in contracting skeletal muscle and unequivocally demonstrated elevated free radical generation [30]–[32]. Bailey et al provided the first direct evidence of exercise-induced free radical accumulation in human muscle [33]. Collectively, there appears a solid foundation to expect a beneficial effect from antioxidant treatment. The absence of protection suggests that nonspecific antioxidants are insufficient to eliminate untoward reactive oxygen species in exercising muscle.

Mitochondria are the predominant contributor of cellular reactive oxygen species [13]. Manganese superoxide dismutase (MnSOD) and glutathione peroxidase (GPX) constitute the primary antioxidant defense system in the mitochondria. Briefly, MnSOD converts superoxide radicals to hydrogen peroxide. Hydrogen peroxide is then reduced by GPX into water. When hydrogen peroxide is not completely detoxified, it readily forms highly reactive and cytotoxic hydroxyl radicals. The reaction catalyzed by GPX highly depends on the availability of reduced glutathione (GSH) to provide electrons. The oxidized glutathione (GSSG) has to be regenerated to GSH in order to maintain GPX activity.

Catalase also breaks down hydrogen peroxide. In contrast to GPX, catalase activity has no cofactor limitation. Catalase is usually expressed in the peroxisome. However, it has also been detected in the mitochondria in rat heart and liver [34]–[36]. Over the last decade, there has been a great interest in experimentally expressing catalase in the mitochondria in the hope of improving cellular defense against free-radical injury [20], [21], [37]–[41]. Collectively, these studies have convincingly demonstrated mitochondrial catalase expression as an effective strategy to ameliorate oxidative damage.

The most striking finding on MCAT is made in transgenic mice. To test the role of oxidative stress in mammalian aging, Schriner et al compared the maximal and median life span in transgenic mice expressing catalase in the peroxisomes, nuclei or mitochondria [20]. They obtained two MCAT transgenic lines with different levels of mosaic MCAT expression. Surprisingly, significant lifespan extension was observed in both MCAT transgenic lines but not in other transgenic mice that expressed catalase in the peroxisome or nucleus. A recent study from a different group further confirmed that overexpressing PCAT is not sufficient to increase mouse lifespan [19]. These results suggest that the subcellular location rather than the absolute amount of total cellular antioxidants is more important in mitigating oxidative stress.

Mitochondria used to be considered as the primary source of muscle free radicals. This view is now questioned (reviewed in [3], [6], [42], [43]). Nevertheless, mitochondria remain an important location of reactive oxygen species production in contracting muscle. Since muscle mitochondria are particularly susceptible to oxidative damage [44], targeted antioxidant delivery to the mitochondria may alleviate oxidative injury in contracting muscle. Promoted by the encouraging results of MCAT transgenic mice, we set out to test whether body-wide delivery of MCAT by an AAV vector can improve exercise performance in normal mice. We have previously shown that AAV-9 is capable of whole body muscle transduction [22], [23], [45]. To this end, we engineered MCAT in an AAV-9 vector and injected into the circulation of newborn BL6 mice. As expected, we observed widespread but mosaic MCAT expression in the heart and skeletal muscle (Figures 1 and 2). Similar to previous reports in MCAT transgenic mice, we did not see any detrimental effect on the growth rate and body weight in AV.RSV.MCAT infected mice (Figure 3) [20], [21], [40]. When challenged with a single bout of exhaustive treadmill running, AV.RSV.MCAT infected mice performed much better irrespective of gender and body weight (Figure 3).

It has been shown that female mice run better than male mice [46], [47]. In our uninfected control mice, we also noticed that the normalized running distance was significantly higher in female mice (p = 0.021). Interestingly, female mice also appeared to respond better to AV.RSV.MCAT treatment. Compared with male mice, AV.RSV.MCAT administration resulted in significantly better improvement in both absolute and normalized running distance in female mice (Figure 3). Taken together, our results suggest that the mitochondria may still represent a critical site of free radical production during exhaustive exercise. Further, targeted expression of catalase to the mitochondria may counteract oxidative muscle damage and enhance performance.

Normal muscle contraction requires low levels of reactive oxygen species (reviewed in [1], [4]). A complete or near-complete elimination of cellular free radicals may affect force production. To determine whether MCAT overexpression compromises basal muscle contraction, we examined the contract profile of the isolated EDL muscle (Figure 4). No difference was observed between AV.RSV.MCAT infected mice and uninfected controls in terms of twitch force, tetanic force, fatigue pattern and eccentric contraction response (Figure 4). We have previously reported a similar observation following AAV-mediated PCAT expression in the EDL muscle [25]. Together, these results suggest that catalase overexpression may not eliminate physiological level (low-level) free radicals inside cell. As a matter of fact, these findings are consistent with the known biochemical property of catalase. In contrast to GPX, catalase has a very low affinity for its substrate when cellular hydrogen peroxide levels are low (reviewed in [6]).

Exercise capacity is influenced by both cardiac function and skeletal muscle activity. Recently, Dai et al reported that MCAT attenuates aging-associated heart function deterioration [21]. Considering the negligible effect of MCAT on the isolated EDL muscle (Figure 4), it appears that cardiac MCAT expression may have played a major role in our observation. Nevertheless, it is still possible that a synergistic effect of MCAT expression in both the heart and skeletal muscle underlies running performance improvement (Figures 1 and 2). Future studies are needed to further define the underlying mechanism(s).

Excessive free radical production has been implicated in aging related mobility reduction and various muscle diseases such as Duchenne muscular dystrophy (reviewed in [2], [48]–[51]). Our results suggest that boosting mitochondrial antioxidant defense with AAV-mediated MCAT expression may help clear out excessive free radicals and reduce oxidative damages under these conditions. The remarkable safety profile of MCAT overexpression seen in this study as well as in MCAT transgenic mice further paves the way to future therapeutic application [20], [21], [40]. Nevertheless, it is important to recognize that moderate levels of free radicals produced during non-exhaustive/regular exercise or supervised exercise (for patients suffering from certain diseases) actually enhance antioxidant defense in the body and provide multi-systemic health benefits.

## Materials and Methods

### Animals

All animal experiments were approved by the Animal Care and Use Committees at the University of Missouri and were in accordance with NIH guidelines. C57BL/6 (BL6) breeders were purchased from Harlan Laboratories, Inc. (www.harlan.com). Neonatal mice used in the study were generated from local breeding colony. All mice were housed in specific-pathogen free animal care facilities and kept under a 12 h light (25 lux)/12 hr dark cycle with free access to food and water. A total of 45 mice were used in the study including 19 male and 26 female mice.

### Recombinant AAV-9 vector production

The mitochondrial tagged human catalase cDNA (MCAT) plasmid (poCAT) was published before [20]. poCAT contains a mitochondria leader sequence from the ornithine transcarbamylase (OTC) gene [52]. In MCAT transgenic mice, transgene expression is directed by the ubiquitous CAG promoter [20]. We designed our AAV vector with the ubiquitous Rouse sarcoma virus (RSV) promoter (Figure 1A). To generate the cis plasmid for AAV packaging (pcisAV.RSV.MCAT), we first amplified the MCAT gene from poCAT using the following pair of the primers. The forward primer was 5′-GCGCGGTACCATGCTGTTTAATCTGAGGATCC-3′. The underlined nucleotides mark the Kpn I site. The reverse primer was 5′-GCGCAAGCTTTCATCCGGACTGCACAAAGGTGTGAATCGC-3′. The underlined nucleotides represent the Hind III site. The PCR product was digested with Kpn I and Hind III and cloned into the Kpn I/Hind III site in pcis.RSV.mcs [53]. The MCAT gene and the cloning junction were confirmed by sequencing.

Recombinant AAV-9 vectors were generated by a triple plasmid transfection protocol described before using pcis.RSV.MCAT, pRep2/Cap9 and pHelper [22], [23], [45]. pRep2/Cap9 encodes AAV replication proteins and AAV-9 capsid (a gift from Dr. James Wilson at the University of Pennsylvania, Philadelphia, PA) [54]. pHelper provides adenoviral helper function (Stratagene, La Jolla, CA). Viral stocks were purified through two rounds of isopycnic CsCl ultracentrifugation followed by dialyzing in HEPES buffer. Viral titer and quality control were performed according to our previously published protocol [22], [23], [55]. The viral titer was 1×10^10^ viral genome particles/µl. The same lot of viral preparation was used for all *in vivo* experiments.

### Systemic AAV-9 delivery in neonatal mice

Facial vein injection was performed in 2-day-old BL6 mice as we described before [22], [23]. A total of 1×10^12^ vg particles of AAV were delivered to each puppy. All AAV injected mice survived the procedure.

### Whole muscle lysate western blot

The freshly isolated muscles were rinsed briefly in 50 mM potassium phosphate buffer (PB), pH 7.8. The muscle was ground to fine powder in a liquid nitrogen cooled mortar with a pestle. The muscle was then homogenized in PB buffer containing 1% protease inhibitor cocktail (Roche, Indianapolis, IN) (10 µl per 1 mg wet muscle weight). The lysate was centrifuged at 10,000 rpm for 5 min at 4°C (Eppendorf centrifuge, model 5417C). The supernatant was collected for western blot. Protein concentration was determined using a Bio-Rad protein assay kit and 50 µg protein/lane was loaded on a 10% SDS-polyacrylamide gel. Catalase was detected with a rabbit polyclonal antibody (1∶1,000; Athens Research & Technology, Athens, GA). Equal loading was confirmed by Ponseu S staining and the intensity of a slow migrating non-specific band [25].

### Mitochondrial preparation western blot

Mitochondria were isolated from the gastrocnemius muscle according to a published protocol with modification [24]. Briefly, the freshly isolated gastrocnemius muscle (∼300 mg) was minced into small pieces and then digested for 30 min at 4°C in 5 ml phosphate buffered saline (PBS) containing 0.2% trypsin, 10 mM EDTA. After a 5 min centrifugation at 200×g, the pellet was resuspended in a buffer containing 50 mM Tris pH 7.4, 50 mM KCl, 10 mM EDTA, 0.2% bovine serum albumin and 67 mM Sucrose. Subsequently, the muscle lysate was homogenized using a tissue tearor (Model 985370-395; Biospec Products Inc. Bartlesville, OK). The homogenate was centrifuged at 700×g for 10 min at 4°C. The supernatant was centrifuged again at 8,000×g for 10 min at 4°C. The pellet was washed in a buffer containing 10 mM Tris pH 7.4, 3 mM EGTA, 250 mM Sucrose. After another round of centrifugation at 8,000×g for 10 min at 4°C, mitochondrial enriched pellet was resuspended in 50 mM PB, pH 7.4. Mitochondrial preparation (70 µg/lane) was resolved in a 10% SDS-polyacrylamide gel and catalase was detected with the Athens' polyclonal antibody as described above. A rabbit polycolonal antibody against prohibitin (1∶1,000; Abcam, Cambridge, MA) was used as the mitochondrial marker in western blot.

### Quantitative muscle catalase activity measurement

Catalase activity in whole muscle lysate was determined using our previously described protocol [25]. Briefly, the liquid nitrogen-snap frozen muscles were pulverized in 50 mM PB, pH 7.8. Crude muscle lysate was further homogenized in a S-3000 sonicator (Misonix Inc. Farmingdale, NY). Cellular debris was then removed by a 5 min centrifugation at 10,000 rpm (Eppendorf centrifuge, model 5417C). Finally, catalase activity in the supernatant was quantified using the Aebi method [56].

### Zymogrphic analysis of muscle catalase activity

In-gel zymography assay was performed as described before in 8% native polyacrylamide gel [25]. Following electrophoresis, catalase was revealed by ferricyanide staining as previously published [57].

### Immunofluorescence staining

Freshly dissected muscle was snap frozen in liquid nitrogen cooled isopentane in the Tissue-Tek OCT compound (Sakura Finetek, Torrance, CA). Eight µm muscle cryosections were fixed in 4% paraformaldehyde for 10 minutes. Catalase was detected with a rabbit polyclonal antibody (1∶500; Calbiochem, San Diego, CA) according to our published protocol [58]. Mitochondria were revealed with a monoclonal antibody against cytochrome C (1∶400; BD Pharmingen, San Jose, CA).

### In vitro analysis of the EDL muscle function

The EDL muscle was carefully isolated from the anesthetized mice and vertically mounted in a jacket organ bath containing oxygenated Ringer's buffer [25]. Muscle twitch force, tetanic force and fatigue response was measured using a 300B dual-mode servomotor transducer (Aurora Scientific, Inc., Aurora, ON, Canada) as we described before [25]. After the fatigue protocol, muscle was allowed a 15 min rest for recovery. We then applied 10 cycles of eccentric contraction stimulation using our published protocol [26].

### Treadmill

Experimental mice were trained on a 15° downhill Exer-3/6 open treadmill (Columbus Instruments, Columbus, OH) for three days. Briefly, on day 1 mice were first placed on an unmoving treadmill for 7 min (2 min flat and 5 min 15° downhill). Mice were then run for 15 min at 5 m/min, 15° downhill. On day 2, mice were first placed on an unmoving 15° downhill treadmill for 2 min. Mice were then run on the 15° downhill for 10 min at 5 m/min followed by another 10 min at 10 m/min. Training on day 3 was similar to that of day 2 except that mice were run at 5 m/min for 5 min and then 10 m/min for 15 min. On the forth day, mice were subjected to a single bout of 15° downhill running starting at the speed of 10 m/min. Twenty min later, treadmill speed was increased at a rate of 1 m/min every 2 min until mice were exhausted. Continuous nudging was used during treadmill to help mice stay on the track. Exhaustion was defined as the point at which mice spent more than 10 sec on the shocker without attempting to resume running when nudged.

### Statistical analysis

Data are presented as mean±standard error of mean (s.e.m.). Statistical analysis was performed with the SPSS software (SPSS, Chicago, IL). Statistical significance between AV.RSV.MCAT infected mice and uninfected mice was determined by student t test. Difference was considered significant when *p*<0.05.
